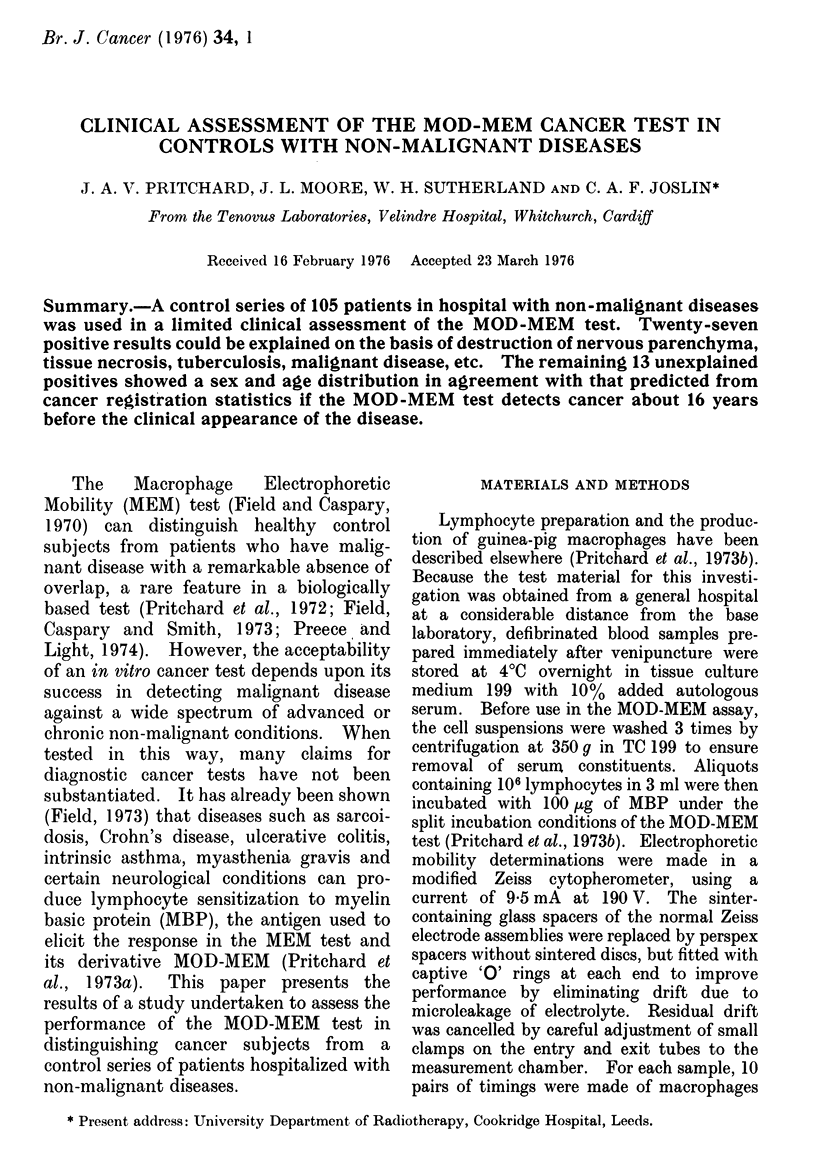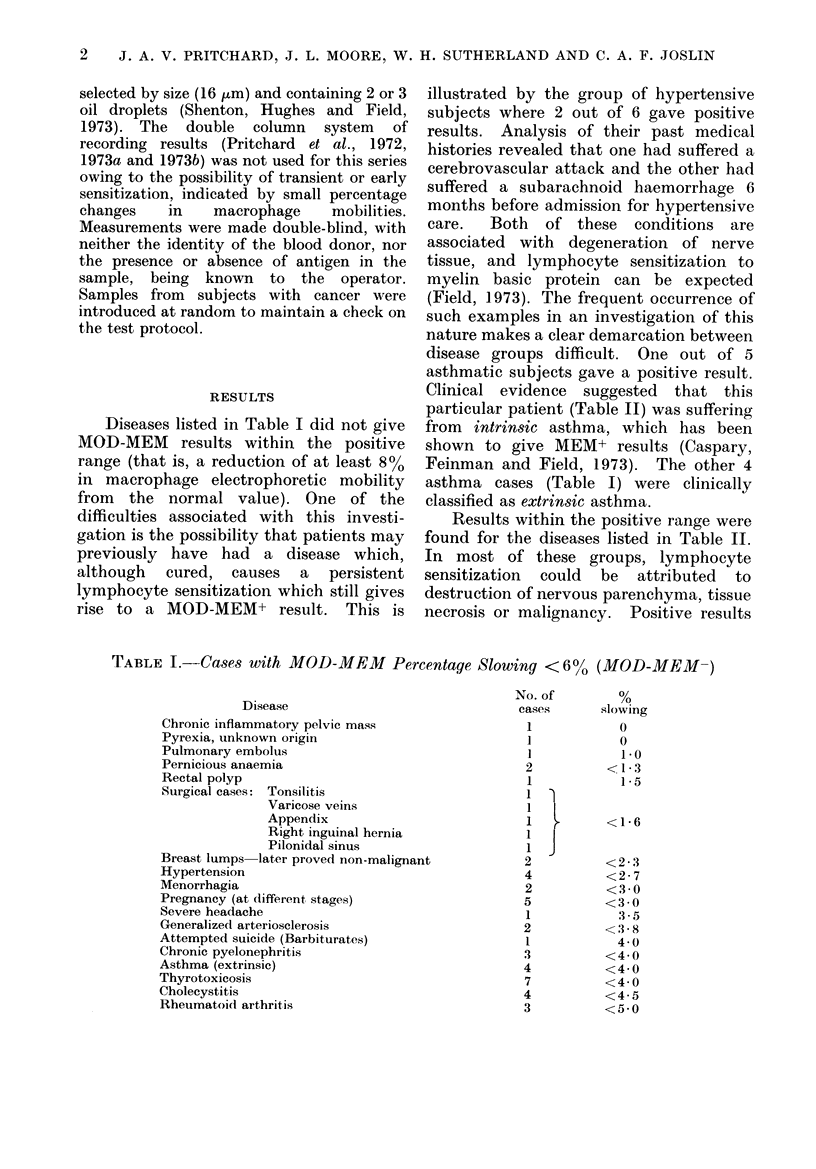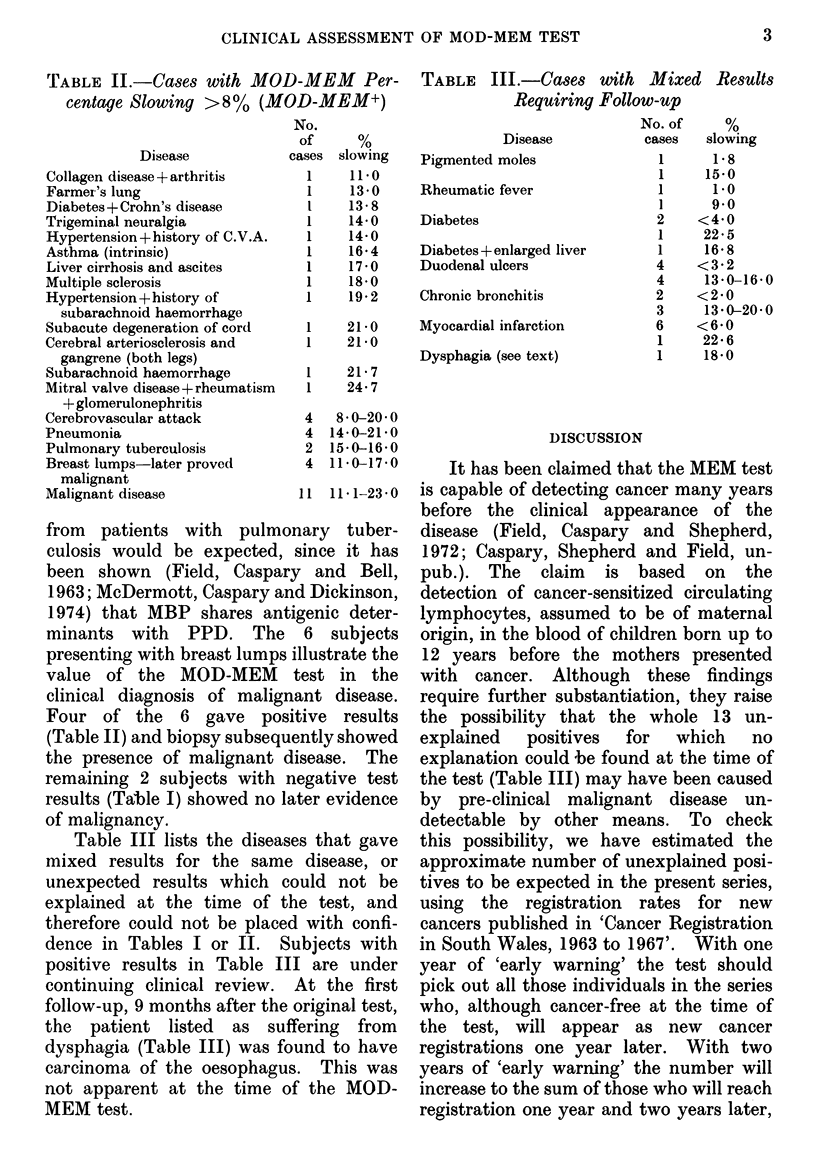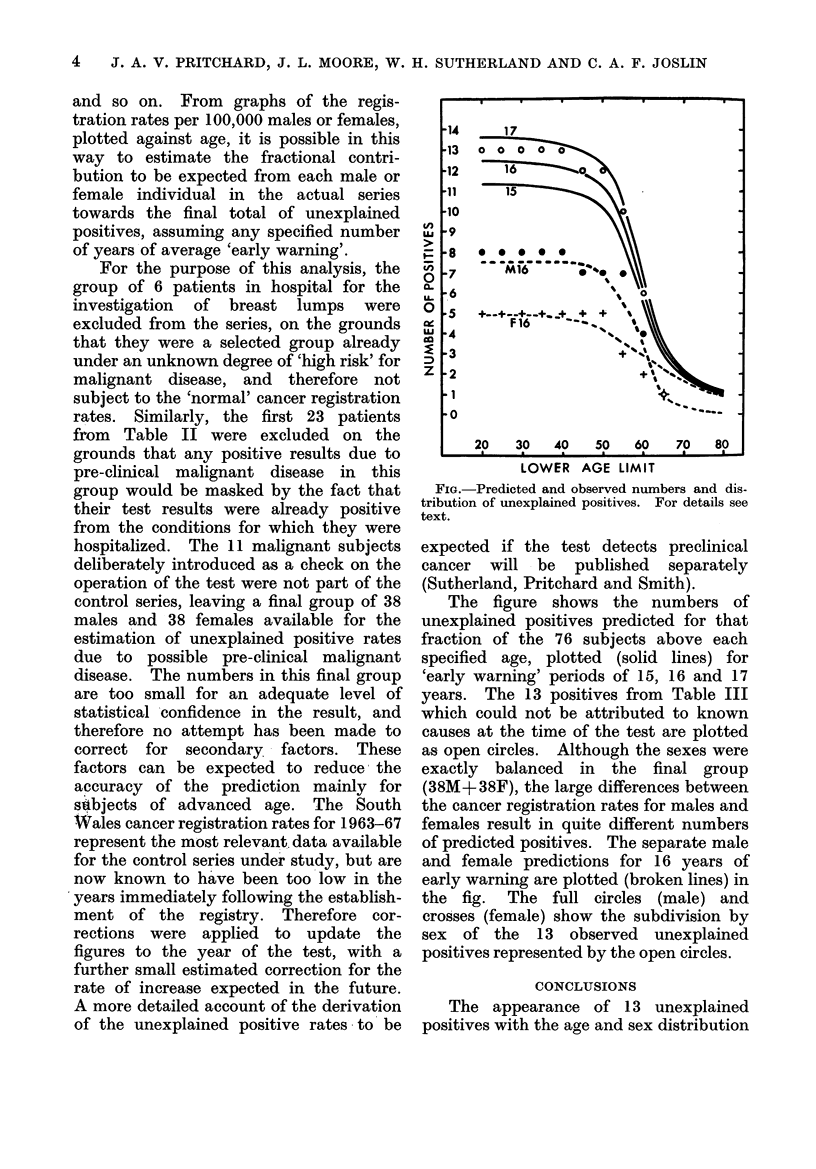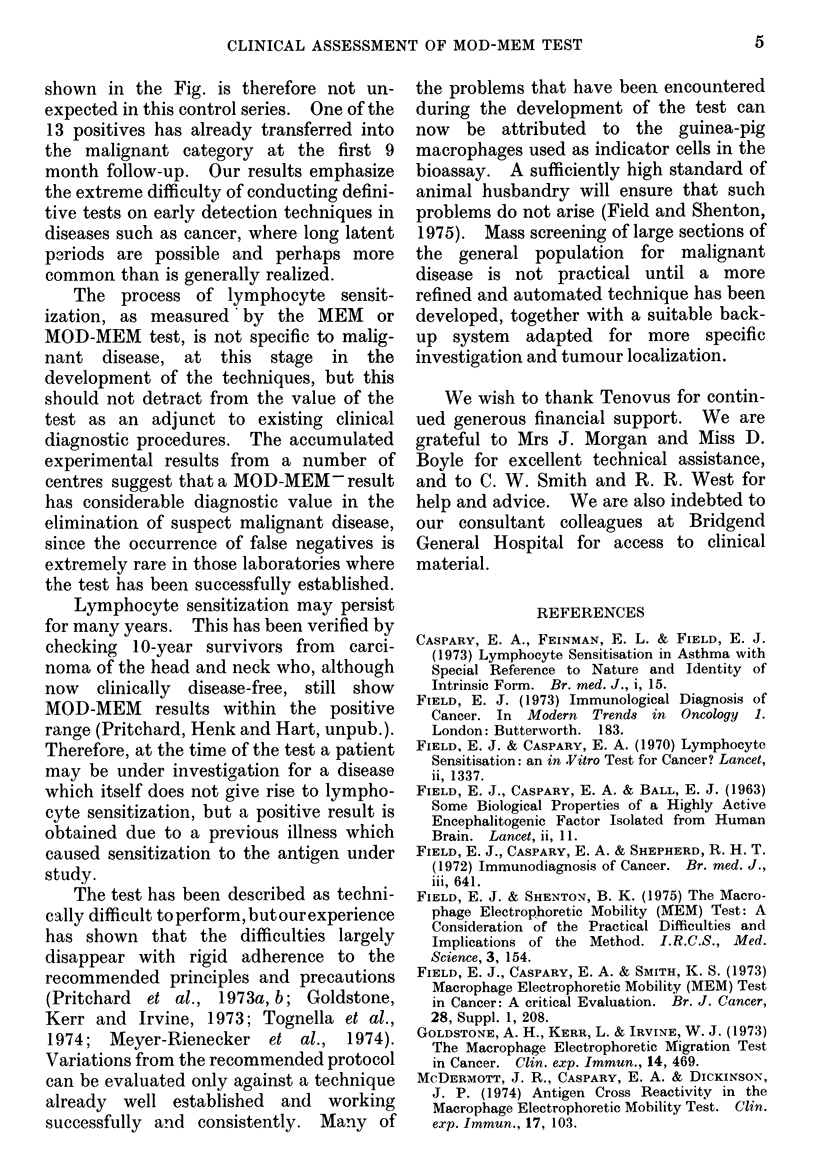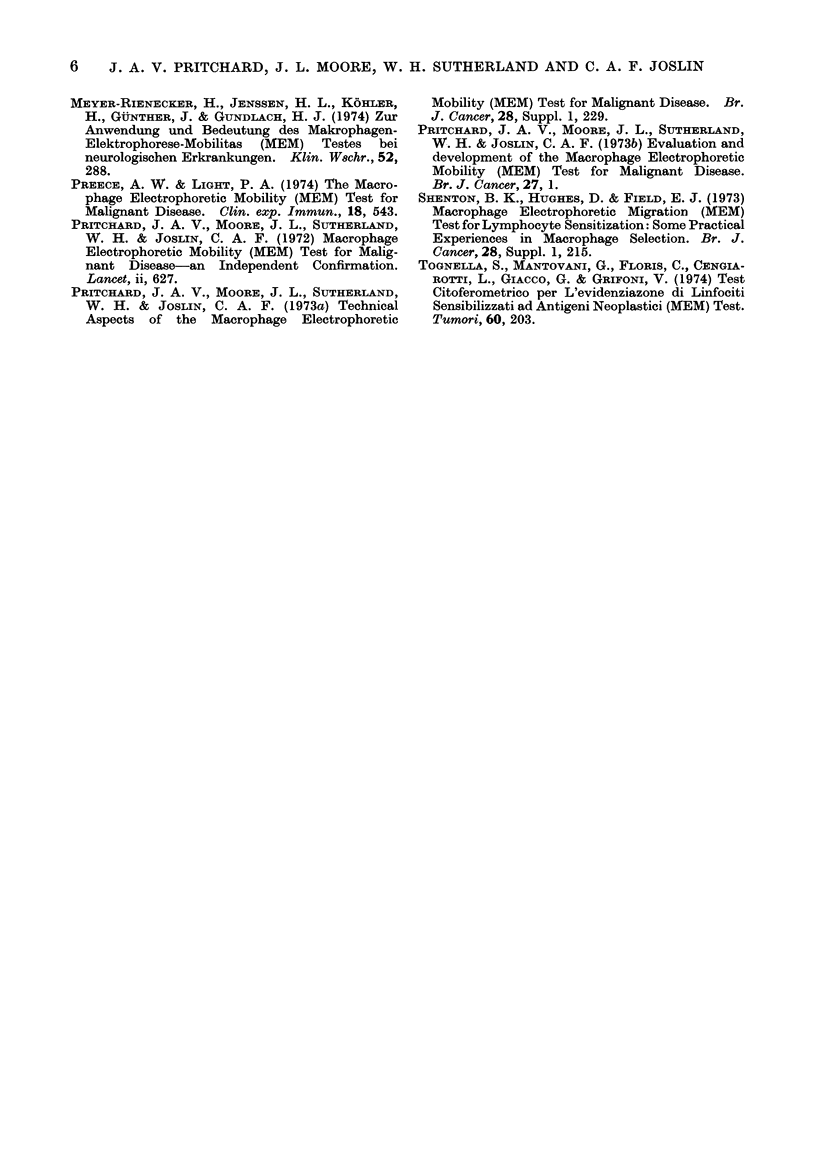# Clinical assessment of the MOD-MEM cancer test in controls with non-malignant diseases.

**DOI:** 10.1038/bjc.1976.113

**Published:** 1976-07

**Authors:** J. A. Pritchard, J. L. Moore, W. H. Sutherland, C. A. Joslin

## Abstract

A control series of 105 patients in hospital with non-malignant diseases was used in a limited clinical assessment of the MOD-MEM test. Twenty-seven positive results could be explained on the basis of destruction of nervous parenchyma, tissue necrosis, tuberculosis, malignant disease, etc. The remaining 13 unexplained positives showed a sex and age distribution in agreement with that predicted from cancer registration statistics if the MOD-MEM test detects cancer about 16 years before the clinical appearance of the disease.


*Br. J. Cancer* (1976) **34**, 1

### CLINICAL ASSESSMENT OF THE MOD-MEM CANCER TEST IN CONTROLS WITH NON-MALIGNANT DISEASES

J. A. V. PRITCHARD, J. L. MOORE, W. H. SUTHERLAND AND C. A. F. JOSLIN*

*From the Tenovus Laboratories, Velindre Hospital, Whitchurch, Cardiff*

Received 16 February 1976 Accepted 23 March 1976

**Summary.—A control series of 105 patients in hospital with non-malignant diseases was used in a limited clinical assessment of the MOD-MEM test. Twenty-seven positive results could be explained on the basis of destruction of nervous parenchyma, tissue necrosis, tuberculosis, malignant disease, etc. The remaining 13 unexplained positives showed a sex and age distribution in agreement with that predicted from cancer registration statistics if the MOD-MEM test detects cancer about 16 years before the clinical appearance of the disease.**

The Macrophage Electrophoretic Mobility (MEM) test (Field and Caspary, 1970) can distinguish healthy control subjects from patients who have malignant disease with a remarkable absence of overlap, a rare feature in a biologically based test (Pritchard *et al.*, 1972; Field, Caspary and Smith, 1973; Preece and Light, 1974). However, the acceptability of an *in vitro* cancer test depends upon its success in detecting malignant disease against a wide spectrum of advanced or chronic non-malignant conditions. When tested in this way, many claims for diagnostic cancer tests have not been substantiated. It has already been shown (Field, 1973) that diseases such as sarcoidosis, Crohn's disease, ulcerative colitis, intrinsic asthma, myasthenia gravis and certain neurological conditions can produce lymphocyte sensitization to myelin basic protein (MBP), the antigen used to elicit the response in the MEM test and its derivative MOD-MEM (Pritchard *et al.*, 1973*a*). This paper presents the results of a study undertaken to assess the performance of the MOD-MEM test in distinguishing cancer subjects from a control series of patients hospitalized with non-malignant diseases.

### MATERIALS AND METHODS

Lymphocyte preparation and the production of guinea-pig macrophages have been described elsewhere (Pritchard *et al.*, 1973*b*). Because the test material for this investigation was obtained from a general hospital at a considerable distance from the base laboratory, defibrinated blood samples prepared immediately after venipuncture were stored at 4°C overnight in tissue culture medium 199 with 10% added autologous serum. Before use in the MOD-MEM assay, the cell suspensions were washed 3 times by centrifugation at 350 *g* in TC 199 to ensure removal of serum constituents. Aliquots containing $10^6$ lymphocytes in 3 ml were then incubated with 100 $\mu$g of MBP under the split incubation conditions of the MOD-MEM test (Pritchard *et al.*, 1973*b*). Electrophoretic mobility determinations were made in a modified Zeiss cytopherometer, using a current of 9·5 mA at 190 V. The sinter-containing glass spacers of the normal Zeiss electrode assemblies were replaced by perspex spacers without sintered discs, but fitted with captive 'O' rings at each end to improve performance by eliminating drift due to microleakage of electrolyte. Residual drift was cancelled by careful adjustment of small clamps on the entry and exit tubes to the measurement chamber. For each sample, 10 pairs of timings were made of macrophages

* Present address: University Department of Radiotherapy, Cookridge Hospital, Leeds.

selected by size (16 μm) and containing 2 or 3 oil droplets (Shenton, Hughes and Field, 1973). The double column system of recording results (Pritchard *et al.*, 1972, 1973*a* and 1973*b*) was not used for this series owing to the possibility of transient or early sensitization, indicated by small percentage changes in macrophage mobilities. Measurements were made double-blind, with neither the identity of the blood donor, nor the presence or absence of antigen in the sample, being known to the operator. Samples from subjects with cancer were introduced at random to maintain a check on the test protocol.

### RESULTS

Diseases listed in Table I did not give MOD-MEM results within the positive range (that is, a reduction of at least 8% in macrophage electrophoretic mobility from the normal value). One of the difficulties associated with this investigation is the possibility that patients may previously have had a disease which, although cured, causes a persistent lymphocyte sensitization which still gives rise to a MOD-MEM$^{+}$ result. This is illustrated by the group of hypertensive subjects where 2 out of 6 gave positive results. Analysis of their past medical histories revealed that one had suffered a cerebrovascular attack and the other had suffered a subarachnoid haemorrhage 6 months before admission for hypertensive care. Both of these conditions are associated with degeneration of nerve tissue, and lymphocyte sensitization to myelin basic protein can be expected (Field, 1973). The frequent occurrence of such examples in an investigation of this nature makes a clear demarcation between disease groups difficult. One out of 5 asthmatic subjects gave a positive result. Clinical evidence suggested that this particular patient (Table II) was suffering from *intrinsic* asthma, which has been shown to give MEM$^{+}$ results (Caspary, Feinman and Field, 1973). The other 4 asthma cases (Table I) were clinically classified as *extrinsic* asthma.

Results within the positive range were found for the diseases listed in Table II. In most of these groups, lymphocyte sensitization could be attributed to destruction of nervous parenchyma, tissue necrosis or malignancy. Positive results

TABLE I.—*Cases with MOD-MEM Percentage Slowing* <6% (*MOD-MEM*$^{-}$)

| Disease | | No. of cases | % slowing |
|---|---|---|---|
| Chronic inflammatory pelvic mass | | 1 | 0 |
| Pyrexia, unknown origin | | 1 | 0 |
| Pulmonary embolus | | 1 | 1·0 |
| Pernicious anaemia | | 2 | <1·3 |
| Rectal polyp | | 1 | 1·5 |
| Surgical cases: | Tonsilitis | 1 | |
| | Varicose veins | 1 | |
| | Appendix | 1 | <1·6 |
| | Right inguinal hernia | 1 | |
| | Pilonidal sinus | 1 | |
| Breast lumps—later proved non-malignant | | 2 | <2·3 |
| Hypertension | | 4 | <2·7 |
| Menorrhagia | | 2 | <3·0 |
| Pregnancy (at different stages) | | 5 | <3·0 |
| Severe headache | | 1 | 3·5 |
| Generalized arteriosclerosis | | 2 | <3·8 |
| Attempted suicide (Barbiturates) | | 1 | 4·0 |
| Chronic pyelonephritis | | 3 | <4·0 |
| Asthma (extrinsic) | | 4 | <4·0 |
| Thyrotoxicosis | | 7 | <4·0 |
| Cholecystitis | | 4 | <4·5 |
| Rheumatoid arthritis | | 3 | <5·0 |

TABLE II.—*Cases with MOD-MEM Percentage Slowing* $>8\%$ (*MOD-MEM*$^+$)

| Disease | No. of cases | % slowing |
|---|---|---|
| Collagen disease + arthritis | 1 | 11·0 |
| Farmer's lung | 1 | 13·0 |
| Diabetes + Crohn's disease | 1 | 13·8 |
| Trigeminal neuralgia | 1 | 14·0 |
| Hypertension + history of C.V.A. | 1 | 14·0 |
| Asthma (intrinsic) | 1 | 16·4 |
| Liver cirrhosis and ascites | 1 | 17·0 |
| Multiple sclerosis | 1 | 18·0 |
| Hypertension + history of subarachnoid haemorrhage | 1 | 19·2 |
| Subacute degeneration of cord | 1 | 21·0 |
| Cerebral arteriosclerosis and gangrene (both legs) | 1 | 21·0 |
| Subarachnoid haemorrhage | 1 | 21·7 |
| Mitral valve disease + rheumatism + glomerulonephritis | 1 | 24·7 |
| Cerebrovascular attack | 4 | 8·0–20·0 |
| Pneumonia | 4 | 14·0–21·0 |
| Pulmonary tuberculosis | 2 | 15·0–16·0 |
| Breast lumps—later proved malignant | 4 | 11·0–17·0 |
| Malignant disease | 11 | 11·1–23·0 |

from patients with pulmonary tuberculosis would be expected, since it has been shown (Field, Caspary and Bell, 1963; McDermott, Caspary and Dickinson, 1974) that MBP shares antigenic determinants with PPD. The 6 subjects presenting with breast lumps illustrate the value of the MOD-MEM test in the clinical diagnosis of malignant disease. Four of the 6 gave positive results (Table II) and biopsy subsequently showed the presence of malignant disease. The remaining 2 subjects with negative test results (Table I) showed no later evidence of malignancy.

Table III lists the diseases that gave mixed results for the same disease, or unexpected results which could not be explained at the time of the test, and therefore could not be placed with confidence in Tables I or II. Subjects with positive results in Table III are under continuing clinical review. At the first follow-up, 9 months after the original test, the patient listed as suffering from dysphagia (Table III) was found to have carcinoma of the oesophagus. This was not apparent at the time of the MOD-MEM test.

TABLE III.—*Cases with Mixed Results Requiring Follow-up*

| Disease | No. of cases | % slowing |
|---|---|---|
| Pigmented moles | 1 | 1·8 |
| | 1 | 15·0 |
| Rheumatic fever | 1 | 1·0 |
| | 1 | 9·0 |
| Diabetes | 2 | <4·0 |
| | 1 | 22·5 |
| Diabetes + enlarged liver | 1 | 16·8 |
| Duodenal ulcers | 4 | <3·2 |
| | 4 | 13·0–16·0 |
| Chronic bronchitis | 2 | <2·0 |
| | 3 | 13·0–20·0 |
| Myocardial infarction | 6 | <6·0 |
| | 1 | 22·6 |
| Dysphagia (see text) | 1 | 18·0 |

### DISCUSSION

It has been claimed that the MEM test is capable of detecting cancer many years before the clinical appearance of the disease (Field, Caspary and Shepherd, 1972; Caspary, Shepherd and Field, unpub.). The claim is based on the detection of cancer-sensitized circulating lymphocytes, assumed to be of maternal origin, in the blood of children born up to 12 years before the mothers presented with cancer. Although these findings require further substantiation, they raise the possibility that the whole 13 unexplained positives for which no explanation could be found at the time of the test (Table III) may have been caused by pre-clinical malignant disease undetectable by other means. To check this possibility, we have estimated the approximate number of unexplained positives to be expected in the present series, using the registration rates for new cancers published in 'Cancer Registration in South Wales, 1963 to 1967'. With one year of 'early warning' the test should pick out all those individuals in the series who, although cancer-free at the time of the test, will appear as new cancer registrations one year later. With two years of 'early warning' the number will increase to the sum of those who will reach registration one year and two years later, and so on. From graphs of the registration rates per 100,000 males or females, plotted against age, it is possible in this way to estimate the fractional contribution to be expected from each male or female individual in the actual series towards the final total of unexplained positives, assuming any specified number of years of average 'early warning'.

For the purpose of this analysis, the group of 6 patients in hospital for the investigation of breast lumps were excluded from the series, on the grounds that they were a selected group already under an unknown degree of 'high risk' for malignant disease, and therefore not subject to the 'normal' cancer registration rates. Similarly, the first 23 patients from Table II were excluded on the grounds that any positive results due to pre-clinical malignant disease in this group would be masked by the fact that their test results were already positive from the conditions for which they were hospitalized. The 11 malignant subjects deliberately introduced as a check on the operation of the test were not part of the control series, leaving a final group of 38 males and 38 females available for the estimation of unexplained positive rates due to possible pre-clinical malignant disease. The numbers in this final group are too small for an adequate level of statistical confidence in the result, and therefore no attempt has been made to correct for secondary factors. These factors can be expected to reduce the accuracy of the prediction mainly for subjects of advanced age. The South Wales cancer registration rates for 1963–67 represent the most relevant data available for the control series under study, but are now known to have been too low in the years immediately following the establishment of the registry. Therefore corrections were applied to update the figures to the year of the test, with a further small estimated correction for the rate of increase expected in the future. A more detailed account of the derivation of the unexplained positive rates to be expected if the test detects preclinical cancer will be published separately (Sutherland, Pritchard and Smith).

FIG.—Predicted and observed numbers and distribution of unexplained positives. For details see text.

The figure shows the numbers of unexplained positives predicted for that fraction of the 76 subjects above each specified age, plotted (solid lines) for 'early warning' periods of 15, 16 and 17 years. The 13 positives from Table III which could not be attributed to known causes at the time of the test are plotted as open circles. Although the sexes were exactly balanced in the final group (38M+38F), the large differences between the cancer registration rates for males and females result in quite different numbers of predicted positives. The separate male and female predictions for 16 years of early warning are plotted (broken lines) in the fig. The full circles (male) and crosses (female) show the subdivision by sex of the 13 observed unexplained positives represented by the open circles.

### CONCLUSIONS

The appearance of 13 unexplained positives with the age and sex distribution shown in the Fig. is therefore not unexpected in this control series. One of the 13 positives has already transferred into the malignant category at the first 9 month follow-up. Our results emphasize the extreme difficulty of conducting definitive tests on early detection techniques in diseases such as cancer, where long latent periods are possible and perhaps more common than is generally realized.

The process of lymphocyte sensitization, as measured by the MEM or MOD-MEM test, is not specific to malignant disease, at this stage in the development of the techniques, but this should not detract from the value of the test as an adjunct to existing clinical diagnostic procedures. The accumulated experimental results from a number of centres suggest that a MOD-MEM$^{-}$ result has considerable diagnostic value in the elimination of suspect malignant disease, since the occurrence of false negatives is extremely rare in those laboratories where the test has been successfully established.

Lymphocyte sensitization may persist for many years. This has been verified by checking 10-year survivors from carcinoma of the head and neck who, although now clinically disease-free, still show MOD-MEM results within the positive range (Pritchard, Henk and Hart, unpub.). Therefore, at the time of the test a patient may be under investigation for a disease which itself does not give rise to lymphocyte sensitization, but a positive result is obtained due to a previous illness which caused sensitization to the antigen under study.

The test has been described as technically difficult to perform, but our experience has shown that the difficulties largely disappear with rigid adherence to the recommended principles and precautions (Pritchard *et al.*, 1973*a*, *b*; Goldstone, Kerr and Irvine, 1973; Tognella *et al.*, 1974; Meyer-Rienecker *et al.*, 1974). Variations from the recommended protocol can be evaluated only against a technique already well established and working successfully and consistently. Many of the problems that have been encountered during the development of the test can now be attributed to the guinea-pig macrophages used as indicator cells in the bioassay. A sufficiently high standard of animal husbandry will ensure that such problems do not arise (Field and Shenton, 1975). Mass screening of large sections of the general population for malignant disease is not practical until a more refined and automated technique has been developed, together with a suitable back-up system adapted for more specific investigation and tumour localization.

We wish to thank Tenovus for continued generous financial support. We are grateful to Mrs J. Morgan and Miss D. Boyle for excellent technical assistance, and to C. W. Smith and R. R. West for help and advice. We are also indebted to our consultant colleagues at Bridgend General Hospital for access to clinical material.

### REFERENCES

CASPARY, E. A., FEINMAN, E. L. & FIELD, E. J. (1973) Lymphocyte Sensitisation in Asthma with Special Reference to Nature and Identity of Intrinsic Form. *Br. med. J.*, i, 15.

FIELD, E. J. (1973) Immunological Diagnosis of Cancer. In *Modern Trends in Oncology 1*. London: Butterworth. 183.

FIELD, E. J. & CASPARY, E. A. (1970) Lymphocyte Sensitisation: an *in Vitro* Test for Cancer? *Lancet*, ii, 1337.

FIELD, E. J., CASPARY, E. A. & BALL, E. J. (1963) Some Biological Properties of a Highly Active Encephalitogenic Factor Isolated from Human Brain. *Lancet*, ii, 11.

FIELD, E. J., CASPARY, E. A. & SHEPHERD, R. H. T. (1972) Immunodiagnosis of Cancer. *Br. med. J.*, iii, 641.

FIELD, E. J. & SHENTON, B. K. (1975) The Macrophage Electrophoretic Mobility (MEM) Test: A Consideration of the Practical Difficulties and Implications of the Method. *I.R.C.S., Med. Science*, **3**, 154.

FIELD, E. J., CASPARY, E. A. & SMITH, K. S. (1973) Macrophage Electrophoretic Mobility (MEM) Test in Cancer: A critical Evaluation. *Br. J. Cancer*, **28**, Suppl. 1, 208.

GOLDSTONE, A. H., KERR, L. & IRVINE, W. J. (1973) The Macrophage Electrophoretic Migration Test in Cancer. *Clin. exp. Immun.*, **14**, 469.

MCDERMOTT, J. R., CASPARY, E. A. & DICKINSON, J. P. (1974) Antigen Cross Reactivity in the Macrophage Electrophoretic Mobility Test. *Clin. exp. Immun.*, **17**, 103.

MEYER-RIENECKER, H., JENSSEN, H. L., KÖHLER, H., GÜNTHER, J. & GUNDLACH, H. J. (1974) Zur Anwendung und Bedeutung des Makrophagen-Elektrophorese-Mobilitas (MEM) Testes bei neurologischen Erkrankungen. *Klin. Wschr.*, **52**, 288.

PREECE, A. W. & LIGHT, P. A. (1974) The Macrophage Electrophoretic Mobility (MEM) Test for Malignant Disease. *Clin. exp. Immun.*, **18**, 543.

PRITCHARD, J. A. V., MOORE, J. L., SUTHERLAND, W. H. & JOSLIN, C. A. F. (1972) Macrophage Electrophoretic Mobility (MEM) Test for Malignant Disease—an Independent Confirmation. *Lancet*, ii, 627.

PRITCHARD, J. A. V., MOORE, J. L., SUTHERLAND, W. H. & JOSLIN, C. A. F. (1973*a*) Technical Aspects of the Macrophage Electrophoretic Mobility (MEM) Test for Malignant Disease. *Br. J. Cancer*, **28**, Suppl. 1, 229.

PRITCHARD, J. A. V., MOORE, J. L., SUTHERLAND, W. H. & JOSLIN, C. A. F. (1973*b*) Evaluation and development of the Macrophage Electrophoretic Mobility (MEM) Test for Malignant Disease. *Br. J. Cancer*, **27**, 1.

SHENTON, B. K., HUGHES, D. & FIELD, E. J. (1973) Macrophage Electrophoretic Migration (MEM) Test for Lymphocyte Sensitization: Some Practical Experiences in Macrophage Selection. *Br. J. Cancer*, **28**, Suppl. 1, 215.

TOGNELLA, S., MANTOVANI, G., FLORIS, C., CENGIAROTTI, L., GIACCO, G. & GRIFONI, V. (1974) Test Citoferometrico per L'evidenziazone di Linfociti Sensibilizzati ad Antigeni Neoplastici (MEM) Test. *Tumori*, **60**, 203.